# Integrated UAV and Satellite Multi-Spectral for Agricultural Drought Monitoring of Winter Wheat in the Seedling Stage

**DOI:** 10.3390/s24175715

**Published:** 2024-09-02

**Authors:** Xiaohui Yang, Feng Gao, Hongwei Yuan, Xiuqing Cao

**Affiliations:** 1 Institute of Farmland lrrigation, Chinese Academy of Agricultural Sciences, Xinxiang 453002, China; yxh@ahwrri.org.cn; 2 Graduate School of Chinese Academy of Agricultural Sciences, Beijing 100081, China; 3 Anhui and Huaihe River Institute of Hydraulic Research, Hefei 230088, China; yuanhongwei@ahwrri.org.cn (H.Y.); cxq@ahwrri.org.cn (X.C.); 4 Anhui Provincial Key Laboratory of Water Science and Intelligent Water Conservancy, Hefei 230088, China

**Keywords:** agricultural drought, soil moisture, Landsat, UAV, XGBoost

## Abstract

Agricultural droughts are a threat to local economies, as they disrupt crops. The monitoring of agricultural droughts is of practical significance for mitigating loss. Even though satellite data have been extensively used in agricultural studies, realizing wide-range, high-resolution, and high-precision agricultural drought monitoring is still difficult. This study combined the high spatial resolution of unmanned aerial vehicle (UAV) remote sensing with the wide-range monitoring capability of Landsat-8 and employed the local average method for upscaling to match the remote sensing images of the UAVs with satellite images. Based on the measured ground data, this study employed two machine learning algorithms, namely, random forest (RF) and eXtreme Gradient Boosting (XGBoost1.5.1), to establish the inversion models for the relative soil moisture. The results showed that the XGBoost model achieved a higher accuracy for different soil depths. For a soil depth of 0–20 cm, the XGBoost model achieved the optimal result (R^2^ = 0.6863; root mean square error (RMSE) = 3.882%). Compared with the corresponding model for soil depth before the upscaling correction, the UAV correction can significantly improve the inversion accuracy of the relative soil moisture according to satellite remote sensing. To conclude, a map of the agricultural drought grade of winter wheat in the Huaibei Plain in China was drawn up.

## 1. Introduction

Drought is a common hydrological phenomenon caused by insufficient rainfall [1] and is characterized by high frequency, long duration, and a wide range of influence [2,3], causing considerable harm to agricultural production and society [4]. The global climate is undergoing changes that are leading to an escalation in the frequency of extreme events such as droughts [5,6]. The mechanism of drought formation is complex, and no universal definition has been established until now [7]. Usually, droughts are divided into meteorological, agricultural, hydrological, economic, and social droughts [8]. Agricultural drought is characterized by soil moisture levels that do not meet the water demand of crops [9]. An agricultural drought is a complex process that is often accompanied by changes in various surface features such as the soil moisture, vegetation water content, canopy temperature, etc. [10]. The monitoring of early-stage agricultural droughts is often based on observations at stations [11]. To monitor large areas affected by droughts, remote sensing imagery has become a common tool [12]. Satellite sensors can detect ground surface information, such as the soil moisture content, vegetation, and the temperature, and conduct wide-range monitoring and evaluations of agricultural droughts [13]. One of the key parameters for monitoring droughts is the soil moisture, which can be estimated by using remote sensing imagery [14]. This method has the advantages of being efficient, fast, and able to cover a wide range of land at a relatively low cost [15].

The most effective remote sensing method involves employing microwave radiation to detect the inversion of the soil moisture change [16]. However, due to a limited spatial or temporal resolution, the inversion of soil moisture may be less accurate, as it can suffer due to interference from radar backscattering coefficients resulting from vegetation, surface roughness, etc. As a result, such a method cannot meet the requirements for the meter-scale monitoring of agricultural drought [17]. Multi-spectral remote sensing satellites can provide high-resolution ground spectral data. However, the inversion method fails to fully account for land surface heterogeneity due to the satellite’s fixed orbit and atmospheric effects, thereby limiting its ability to achieve simultaneous wide-range, high-resolution, and high-precision agricultural drought monitoring [15]. Unmanned aerial vehicle (UAV) imaging, one of the low-altitude remote sensing technologies, allows for the fine-grained acquisition of field information, such as crop growth, soil moisture levels, diseases and insect pests, drought stress, etc. [18,19,20,21]. The utilization of UAV technology to monitor the soil moisture content and crop water conditions and to detect crop drought stress represents an effective approach towards achieving precise agricultural drought surveillance on farmland. Research indicates that the accuracy of soil moisture obtained from the inversion based on UAV remote sensing is usually higher than that based on satellite remote sensing [22,23,24,25,26,27]. UAV-based methods for soil moisture monitoring include the band reflectance method [24] and the spectral index method [25,26,27]. Feng et al. (2020) [25] utilized the perpendicular drought index (PDI) to carry out soil moisture inversions on a farmland scale. The model coefficient of determination, R^2^, was greater than 0.8, while the root mean square error, RMSE, was less than 0.1, which confirmed the feasibility of this soil moisture monitoring method for farmland based on UAV images. Zhu et al. (2023) [26] employed a suite of sensitive vegetation indexes (including the CARI, TCARI2, SAVI, and OSAVI, among others) in conjunction with random forest (RF), support vector machine (SVM), and extreme learning machine for regression and multiclass classification (ELM) models to simulate kiwi root-zone soil moisture (SM). Their models demonstrated a strong predictive performance for the shallow root-zone SM, with R² values ranging from 0.65 to 0.82 and an RMSE spanning 0.02 to 0.03. These findings underscore the remarkable potential of integrating machine learning (ML) methodologies into unmanned aerial vehicle (UAV)-based SM estimations.

Even though wide-range macroscopic monitoring can be achieved using satellite images, UAV-based fine-resolution images should be also implemented in order to determine better the heterogeneity of the actual land surface and reduce the gap between field measurements and satellite data [28,29]. Therefore, the best choice is to combine the advantages of these two methods so as to achieve quickly the wide-range and high-precision monitoring of surface features and obtain data. The feasibility of the combination of satellite and UAV imaging for agricultural monitoring has been verified by multiple scholars. Zhang et al. (2019) [30] used satellite, UAV and ground data to invert the SPAD of winter wheat during the period of seedling establishment. Xi et al. (2019) [31] improved the inversion of soil salinity in the winter wheat planting area based on Sentinel satellite and UAV multi-spectral remote sensing data. Zhao et al. (2019) [32] combined data from Sentinel-2A with the data from a UAV to improve the accuracy of crop classification. Mazzia et al. (2020) [33] employed high-resolution UAV image information to refine a satellite-based NDVI graph. Jiang et al. (2023) [34] corrected a satellite-based estimation model for wheat growth and nitrogen content based on UAV images. Thus, the combination of UAV and satellite data can be used to improve the accuracy when extracting surface feature information. However, at the present time, only a small amount of research combining satellite and UAV technologies has focused on agricultural drought monitoring.

The objective of this research was twofold: to analyze changes in the agricultural drought affecting wheat in the Huai River Basin in China, and to determine the accuracy of satellite UAV technologies in detecting variations in soil moisture.

The Huai River Basin in China is an important commodity grain production base, as well as an area that suffers from frequent droughts [35]. Extreme events such as droughts have become more frequent and affect the winter wheat crops in the Huaibei Plain of Anhui Province [36]. Therefore, it is important to increase the amount of research on efficient ways to monitor agricultural droughts, as they have a direct economic impact on this region. This study used the winter wheat in the Huaibei Plain of Anhui Province as the research subject, and combined the advantages of satellite and UAV imagery to improve the accuracy of inversion soil moisture and draw a high-resolution and high-precision map of agricultural droughts.

## 2. Materials and Methods

### 2.1. Research Area and Observational Data

**Figure 1:**
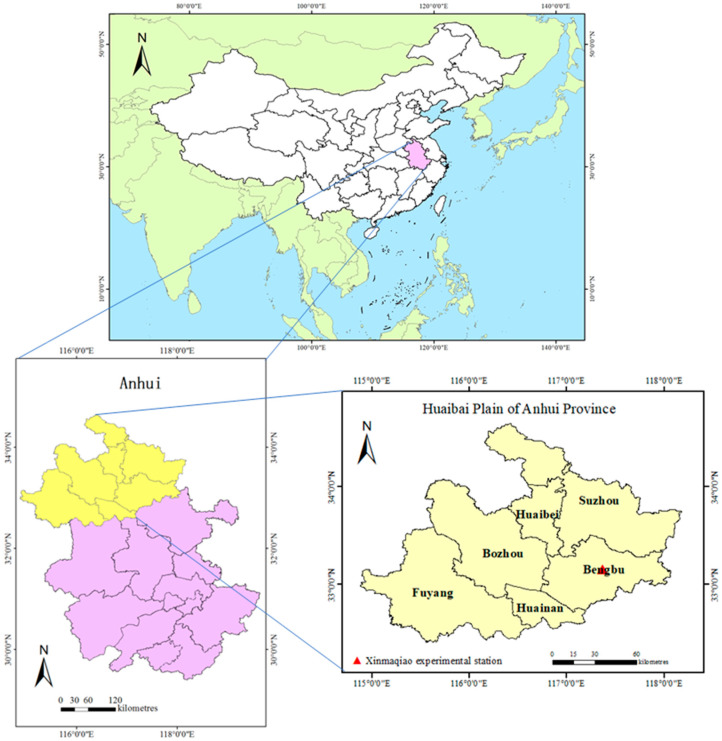
Schematic of the study area.

The Huaibei Plain in Anhui Province is located in the south of the Huang-Huai-Hai Plain (Figure 1). The climate is northern subtropical with warm temperatures and a semi-humid monsoon climate, characterized by dry winters with little rainfall and hot summers with abundant rainfall [37]. The mean annual precipitation is 850 mm, the mean annual temperature is 14.7 °C, and the frost-free period lasts for more than 206 days [38]. The inter-annual and intra-annual distribution of precipitation is uneven, with great inter-annual variations, causing frequent agricultural droughts that directly impact the growth of the winter wheat in the Huaibei Plain. The droughts have a catastrophic impact on the crops and the overall regional economy as the Huaibei Plain is one of the main wheat-producing areas in China, and winter wheat is the main grain crop in such areas [37,39].

**Figure 2:**
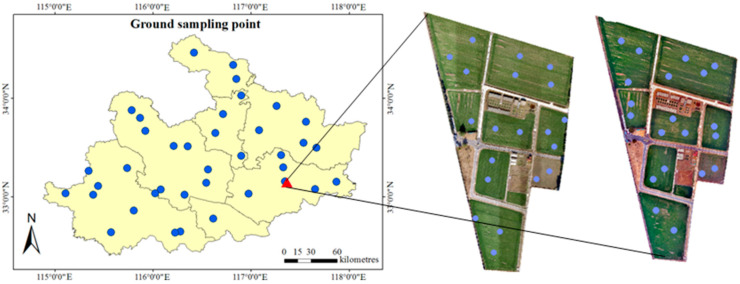
Schematic diagram of measured sampling points.

The soil moisture data were obtained from the observational data on the soil moisture in Huaihe River Basin. The soil moisture was sampled using a soil auger once every 10 days. The weight moisture content of each soil layer was determined using the drying method. The field water-holding capacity and wilting water content of each soil layer were obtained from the experimental report of the station. The soil moisture data at different depths of the 38 stations (Figure 2) were standardized and calculated as the relative soil moisture content of each soil layer. The depths of the soil observations were set to 0–10 cm, 10–20 cm, and 20–40 cm, and 76 sets of relative soil moisture (RSM) were recorded from 21 November 2021 to 1 December 2021.

The UAV observations were conducted at the Xinmaqiao Comprehensive Agricultural Water Conservancy and Irrigation Test Station. The test station is located in Guzhen County, north of Bengbu, at an altitude of 19.70 m (33°09′ N, 117°22′ E). The soil type in the test area is lime concretion black soil, and the soil texture is clay loam, which is the main soil type in the area [40]. The geographical environment, natural conditions, and crop cultivation at the test station are representative of the Huaibei Plain [41]. The UAV imagery and manual sampling of the soil were carried out at the same time. In total, 20 manual soil-sampling points (40 groups) were arranged on the ground (Figure 2), and the observation depths of the soil were set to 0–10 cm, 10–20 cm, and 20–40 cm, respectively. According to the data of the stratified field capacity at the test station, the measured values were calculated as the relative soil moisture (RSM). During the selection of sampling points on the ground, a Trimble GEO 7X centimeter hand-held GPS instrument was used to calibrate the longitude and the latitude in order to obtain the information of corresponding points on the remote sensing images.

### 2.2. Acquisition and Processing of Remote Sensing Data

#### 2.2.1. Satellite Remote Sensing Data

The remote sensing data were obtained from the Landsat-8 satellite (https://earthexplorer.usgs.gov/) (accessed on 21 November 2023). Due to limits provided by the presence of clouds and other weather-related limitations on the imagery, which could impede a clean analysis of the images, this study used only 2 scenes, taken during the seedling stage of the winter wheat in 2021: on 19 November 2021 and 5 December 2021. The downloaded data were Collection 2 Level 2 images, for which an atmospheric radiation correction and a radiometric calibration were performed. The main bands used included b2, b3, b4, and b5, corresponding to blue (B), green (G), red (R), and near-infrared (NIR), respectively; the spectral coverage was 0.45–0.51 μm, 0.53–0.59 μm, 0.64–0.67 μm, and 0.85–0.88 μm, respectively; and the resolution was 30 m. During this study, the ENVI 5.3 software was used for the mosaic cutting of data and reflectance extraction.

#### 2.2.2. UAV Multi-Spectral Remote Sensing Data

A DJI Phantom 4 (RTK) multi-spectral UAV(Da Jiang Innovations, Shenzhen, China) was used. It had 5 bands: b1, b2, b3, b4, and b5, corresponding to blue (B), green (G), red (R), red edge (RE), and near-infrared (NIR). The central wavelengths were 450 nm, 560 nm, 650 nm, 730 nm, and 840 nm, respectively. According to the transit time of Landsat-8 above the test station, two groups of UAV multi-spectral images were obtained on 12 November 2021 and 28 November 2021. The UAV multi-spectral data were collected on sunny days without wind, the flight altitude was 100 m, both the longitudinal overlap and the lateral overlap were 80%, and the ground resolution was 5.3 cm/pixel. Before the UAV multi-spectral images were obtained, a diffusion reflection plate (with a reflection efficiency of 36% and a size of 0.5 m × 0.5 m) was arranged in the flight area to calibrate the pixel brightness value of the multi-spectral images (digital number, DN). The lens of the multi-spectral camera used a nadir view. The high-precision GNSS mobile station D-RTK 2 (DJI) (Da Jiang Innovations, Shenzhen, China)was used. The UAV multi-spectral data were calibrated and synthesized using the DJI Terra (v3.3.4) software (http://www.dji.com/dji-terra) (accessed on 21 November 2022) and stored in TIF format. Finally, the ENVI software was used for data processing and reflectance extraction.

### 2.3. Spectral Index

For agricultural drought monitoring based on optical remote sensing, several researchers calculated the spectral indexes that are sensitive to the surface moisture content, so as to reflect the status of agricultural drought. Shahzad et al. (2019) [42] analyzed the correlation between droughts and climate using the normalized difference vegetation index (NDVI) and rainfall data. Ali et al. (2007) [43] proposed the perpendicular drought index (PDI) according to the spatial features of the spectrum within the infrared–near-infrared band, which can be used to effectively monitor the drought conditions in bare-soil areas. Hazaymeh et al. (2017) [44] combined the normalized difference water index (NDWI), the visible light–shortwave drought index (VSDI), and the land surface temperature (LST) together to build a model, which was used to monitor agricultural droughts in semi-arid regions. Li Xiang and Jianli (2015) [45] carried out a soil water content inversion based on the HJ-1A HSI hyperspectral indexes and confirmed that their model was best for soil depths of 0–10 cm, and that the optimal combination of spectral indexes was the atmospheric resistance vegetation index (ARVI), the ratio vegetation index (RVI), the improvement of simple ratio index (MSR), the normalized difference vegetation index (NDVI), and the optimized soil adjusted vegetation index (OSAVI).

This study selected 4 bands, i.e., blue (B), green (G), red (R), and near-infrared (NIR), to facilitate a UAV-based satellite model correction and the calculation of spectral indexes. B, G, R, and NIR are bands shared by both satellites and UAVs. Meanwhile, this study selected 10 remote sensing indexes for monitoring agricultural drought, which had been verified in relevant references, as shown in Table 1.

### 2.4. Model Building and Precision Evaluation

Random forest (RF) and eXtreme Gradient Boosting (XGBoost) packages were employed in the Python (v3.7) software to build a machine learning model. The coefficient of determination (R^2^) and root mean square error (RMSE) were used to evaluate the model’s precision. When R^2^ approaches 1, the RMSE becomes smaller, indicating a higher model precision.

RF is an integrated learning algorithm. This method is typically used with ensembles of DTs, which are combined in a way that allows the algorithm to fit complex, non-linear relationships between inputs and outputs by combining the predictions of multiple DTs. An RF model is a commonly utilized tool in soil moisture inversions. The model has demonstrated efficacy in root-zone soil moisture inversions, which is the focus of this investigation [26,46]. Thus, RF was employed to conduct soil moisture inversions at varying depths and for agricultural drought research in the study of this paper.

XGBoost uses the second-order Taylor expansion to approximate the loss function and obtains the optimal tree structure and leaf node value by minimizing the loss function. Its basic principle is to establish multiple weak learners based on complete data and aggregate all of the modeling results, so as to obtain a better regression or classification performance. The most important characteristic of such an algorithm is that it can realize accelerated computing through learning, thus improving the accuracy by optimizing the objective function [47]. The XGBoost model was demonstrated in several academic studies to outperform the RF (Random forest), SVR (Support Vector Regression), and PLSR (Partial Least Squares Regression) models in soil moisture inversions [19,48].

### 2.5. Correction of Satellite Image Reflectance

Given the discrepancy in the spectral resolution between the satellite and UAV data, it was necessary to correct the spectral band of the satellite data. Prior research demonstrated the viability of UAV upscaling for satellite applications through the utilization of a local average re-sampling technique, as evidenced in [31,32,33,34]. The local average method is primarily employed for the purpose of upscaling transformations. The averaging of filtering operations allows for the reflection of mean characteristics within the local scope. In particular, the spectral reflectance of a single pixel within each band of the UAV upscaling image and the spectral reflectance of a single pixel of the satellite within the corresponding band are extracted. The mean value of the band ratio method is then employed to determine the correction factor for the four bands of the satellite. The correction factor for each band is multiplied by the parameter of the corresponding satellite band, thus correcting the spectral independent variables that have been screened from the model.

## 3. Results

### 3.1. Analysis of the Correlation between Spectral Band and Remote Sensing Indexes

**Figure 3:**
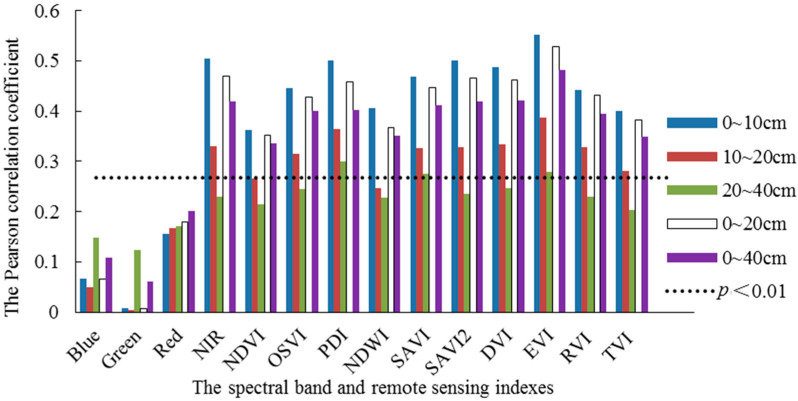
Pearson correlation coefficient of band and spectral indexes and soil moisture content at different depths.

The Pearson correlation between the band reflectance and the agricultural drought indexes of the Landsat remote sensing data and the measured relative soil moisture was analyzed, and a preliminary analysis of the degree of correlation between the relative soil moisture and different spectral indexes was performed. The results in Figure 3 show that the relative soil moisture at a depth of 0–20 cm was the mean value of the relative soil moisture at depths of 0–10 cm and 10–20 cm, and the relative soil moisture at a depth of 0–40 cm was the mean value of the relative soil moisture at depths of 0–20 cm and 20–40 cm. Significance tests were conducted by referring to the table of critical values for correlation coefficient tests, and individual abnormal data, i.e., negative reflectance and spectral indexes were eliminated. The number of sample data points was 92 and the corresponding degree of freedom was 90. When the absolute value of the coefficient of correlation was greater than 0.267, the significance level reached 0.01. As shown in Figure 3, among four bands, the relative soil moisture at the depths of 0–10 cm, 10–20 cm, 0–20 cm, and 0–40 cm only reached the significance level within the NIR band. The near-infrared band was within the high-reflectance area of the crop, which is commonly used for crop identification and has a strong correlation with soil moisture. Therefore, there was a strong correlation between spectral indexes such as the PDI, SAVI, SAVI2, and EVI established on the basis of this band and the relative soil moisture in these four soil layers. Compared with the other depths, the coefficient of correlation between the soil moisture content at a depth of 20–40 cm and each band and spectral index was the smallest, and only the correlations with the PDI and EVI were significant. These results are consistent with the research conclusions in [49]. The possible reason for this is that, as the soil depth increases, the capability of remote sensing inversion declines.

### 3.2. Inversion Model for Relative Soil Moisture at Different Depths Based on UAV Upscaling Correction

Four bands and ten spectral indexes were used as characteristic values and were input into the model. Two kinds of learning algorithms (XGBost and RF) were used for the inversion of the relative soil moisture at different depths, and R^2^ and the RMSE were used to compare the accuracy of the models. The results are shown in Table 2.

From the results of the model training with the two algorithms, we can see that, according to the comparative analysis of the predicted values and actual values of the relative soil moisture at different depths before the upscaling correction, both models were able to invert the surface soil moisture at a depth of 0–20 cm. Compared with the model for soil layers at other depths, they had the highest determination coefficient R^2^ (0.460 and 0.551, respectively) and their RSMEs were 5.253% and 4.803%, respectively. The relative soil moisture at a depth of 10–20 cm ranked second, with the determination coefficient R^2^ of the two models being 0.451 and 0.500 and their RSMEs being 5.452% and 5.128%, respectively. These values were higher than those in other soil layers. The determination coefficients of the two models for the relative soil moisture at a depth of 20–40 cm were the lowest (0.385 and 0.451, respectively), and their RSMEs were 5.889% and 5.498%, respectively. According to the comparison of the inversion capability of the two models for different soil layers before the upscaling correction, the XGBoost model had a higher R^2^ and a smaller RSME, and it was better able to predict the relative soil moisture at different depths.

After the upscaling correction, RF and XGBoost still had the highest determination coefficients (R^2^) for the surface soil moisture at a depth of 0–20 cm, which were 0.540 and 0.686, respectively, and their RSMEs were 4.4246% and 3.8823%, respectively. The R^2^ values of the two models were more statistically significant compared to those before the upscaling correction, by 0.080 and 0.134, respectively. Additionally, their RSMEs declined. The relative soil moisture at a depth of 10–20 cm ranked second; the determination coefficients R^2^ of the two models were 0.503 and 0.640 and their RSMEs were 4.5925% and 4.0134%, respectively. Thus, the R^2^ of the two models improved by 0.052 and 0.139 compared with those before the upscaling correction. The inversion precision of the two models for different soil depths significantly improved after the upscaling correction. There was a certain reduction in the RMSE, indicating that the upscaling correction can improve the inversion precision of satellite remote sensing for relative soil moisture. This analysis suggested that the local average method should be used for pixel binning and the mean value of the pixels should be used as the value of the new pixel after binning, which could reduce the influence of an uneven division between the modeling set and the validation set to a certain degree [34]. After the upscaling correction, XGBoost had a better inversion ability for soil layers at different depths compared to RF. The XGBoost algorithm is a decision tree-integrated algorithm that can be used to solve classification and regression problems, capturing the complex interactions and non-linear relations between variables. In addition, it can reduce the model variance and prevent overfitting, simplifying the model [50].

### 3.3. Agricultural Drought Grade of Winter Wheat during Seedling Stage in the Huaibei Plain of Anhui Province

In this research area, the distribution map of winter wheat crops was drafted on the basis of the 2020 Land Use Type Map of Anhui Province, with a resolution of 30 m (https://zenodo.org/record/5816591) (accessed on 19 August 2021) [51], by visually interpreting the NDVI thresholds. The area of winter wheat planting areas was compared with the data in the Statistical Yearbook of Anhui Province. The error was less than 10%, so it could be used for further study.

On the basis of the Chinese national standard Grade of Agricultural Drought (GB/T 32136—2015) [52], the RSM was used for the classification of agricultural drought in this study. For clay soil, when the RSM was less than or equal to 55 and less than 65, the drought was classified as light; when 45 ≤ RSM < 55, it was classified as a moderate drought; when 35 ≤ RSM < 45, it was classified as a severe drought; and when the RSM was less than 35, the drought was classified as excessive. 

**Figure 4:**
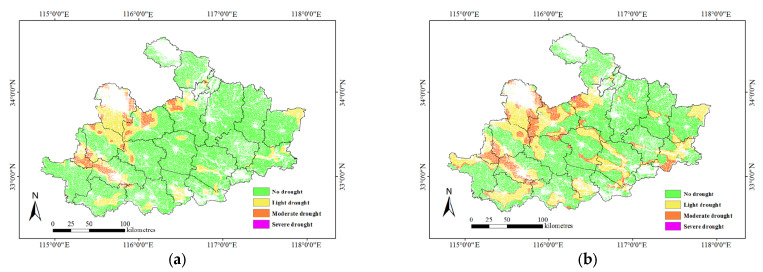
Agricultural drought grade distribution map of Huaibei Plain in Anhui Province based on model inversion. (**a**) November, (**b**) December.

A soil depth of 0–20 cm was used for the inversion of relative soil moisture during the sowing stage and the seedling stage. For an upscaling correction with a soil depth of 0–20 cm, the XGBoost model had a better inversion effect. Therefore, this model was used for the inversion of the relative soil moisture in the Huaibei Plain in November and December, and the agricultural drought was classified according to the national standard, as shown in Figure 4.

According to the results of the model inversion, droughts occurred in the Huaibei Plain in the middle ten days and last ten days of November 2021. Specifically, moderate droughts and severe droughts mainly occurred in northwestern Anhui, including Bozhou City, Fuyang City, Huaibei City, and Bengbu City; severe droughts mainly occurred in Fuyang City, Jieshou City, Taihe County, Guoyang County, and Suixi County; and no droughts occurred in the other areas. After December, less rainfall made the droughts more serious, and the area of moderate drought and severe drought increased. In addition, the drought-stricken area in northwestern Anhui Province expanded, and certain droughts occurred in the southeastern part of Anhui Province. The severe droughts were mainly distributed in Fuyang City, Jieshou City, Taihe County, Guoyuang County, Suixi County, and Wuhe County. According to the rainfall analysis, the cumulative rainfall in most areas within this period was less than 5 mm. Two reasons can be identified to explain the small amount of water: (1) the shortage of rain and the long-lasting and continuous sunny days during this period of the year, and (2) the continuous growth of winter wheat, which reduced the soil moisture and aggravated the drought. Overall, the model accurately pointed out the grade and spatial distribution of droughts in the Huaibei Plain in Anhui Province, and it effectively reflected the degree and variation trend of agricultural droughts in the research area.

## 4. Discussion

### 4.1. Research Model and Accuracy

The model and precision of agricultural drought monitoring based on soil moisture are the primary areas of focus in extensive research. Restricted by the complexity of interactions between different soil types and plants, the precision of inversion based on soil moisture is limited. Several scholars have conducted research on agricultural droughts based on relative soil moisture. Song et al. (2017) [49] used three kinds of remote sensing drought indexes (apparent thermal inertia (ATI), anomalies of vegetation index (AVI), and vegetation supply water index (VSWI)) to invert the relative soil moisture in northwestern Liaoning Province in China. The study results showed an R^2^ = 0.492 for the surface soil (0~10 cm). Sun et al. (2010) [53] selected the temperature vegetation dryness index (TVDI) and the vegetation supply water index (VSWI) to build a soil-moisture-based model for the remote sensing drought monitoring of winter wheat in the main producing areas in China. In this case, the correlation (*p* < 0.01) between the TVDI and the soil moisture at a 10 cm depth reached 0.37 during the mature period of winter wheat. Tong et al. (2020) [54] combined the vegetation index with the evapotranspiration index to establish a drought severity index (DSI), and a quantitative evaluation was conducted to determine the applicability of the DSI to drought monitoring in Shandong Province. The coefficient of correlation between the RSM (0~20 cm) of summer maize and the DSI was 0.37. This study differs from previous research because it implemented a combination of data collection technology to establish with higher accuracy soil moisture.

The generation of soil moisture maps with a high spatial and temporal resolution is a prerequisite for the effective monitoring of agricultural droughts, the management of irrigation, the monitoring of yields, and other related activities. The utilization of high-resolution and freely accessible satellite data can facilitate the advancement of precision soil moisture monitoring. Monteiro et al. (2024) [55] modelled the vegetation and soil moisture content with Sentinel-1 and -2 satellite data. The results revealed where the link with predictors decreases from the early summer (R^2^ = 0.33, RMSEcv = 16.0%) and mid-summer (R^2^ = 0.30, RMSEcv = 17.8%; based on S2_NDMI) to the end of summer (non-significant). Luo et al. (2024) [56] improved the robust extreme learning machine (RELM) algorithm, which had the best effect for the inversion of the soil moisture content in the surface layer (0–20 cm), with the validation set R^2^_adj_ of 0.696 and an RMSE of 0.018. The inversion results of different model data in different regions are different. Additionally, soil moisture inversion models based on satellites encompass a range of models, from linear to non-linear, from single-indicator inversion to multi-indicator joint inversion, and from single-spectrum to multi-spectrum, thermal infrared, microwave, and other joint inversions. In this study, the soil moisture inversion of Huaibei Plain with a high precision was obtained by using a multi-index nonlinear model.

### 4.2. Integrated UAV and Satellite

The reduction in their cost has led to a significant increase in the use of drones in agricultural production. Unmanned aerial vehicles (UAVs) are frequently deployed for field observations, either independently or in conjunction with satellite data that may be difficult to obtain. Now, the remote sensing capabilities of unmanned aerial vehicles (UAVs) have been markedly enhanced, enabling the capture of images with a resolution of up to one centimeter. Furthermore, UAVs possess the flexibility to interchange lenses (multispectral, thermal infrared, LiDAR) according to the research requirements and regulate their flight paths. The necessity for unmanned aerial vehicles (UAVs) to assume a more prominent role in regional agricultural management calls for an examination of the potential for synergistic utilization in conjunction with satellites. The combination of satellite sensors (Landsat, MODIS, Sentinel, etc.) [55,57,58] and spectral bands (such as multispectral, thermal infrared, and microwave, etc.) [49,53,58] is a more prevalent phenomenon, exemplifying the potential for synergistic outcomes. Nevertheless, the considerable potential of satellite–drone synergy appears to be currently underutilized [59]. Emilien et al. (2021) [60] categorized the level of coordination between drones and satellites as follows: “data comparison”, “multiscale explanation”, “model calibration”, and “data fusion”. Currently, the ”model calibration“ mode is used more in the applied research of precision agriculture based on chlorophyll inversion, soil salinity inversion, nitrogen content monitoring, etc. [30,31,34], which is based on the synergistic use of unmanned aerial vehicles (UAVs) and satellites. However, there is a paucity of studies that have integrated satellite and UAV data in the context of agricultural drought.

The scale transformation of remote sensing images represents a significant challenge in the field of geographical research. The existing remote sensing data are of a varying spatial resolution. The discrepancies between the data sets result in errors in the representation of the same object, which in turn affects the outcomes of feature extraction, the spatial pattern analysis, and the quantitative inversion of the object [61,62]. Hassan-Esfahani et al. (2017) [63] proposed a new variational downscaling scheme that utilizes the synchronized aerial image product of the unmanned aerial system, designated “AggieAir”. The results, both quantitative and qualitative, demonstrate that the downscaling method can effectively enhance the spatial resolution of Landsat images by a factor of two to four orders of magnitude. The findings of this research regarding the scale transformation of remote sensing images can provide theoretical and technical support for the retrieval of product authenticity. Overall, the methodology applied in this study helped to solve the problem of the low monitoring precision of satellite imagery, which has significant research and practical application value.

### 4.3. Agricultural Drought Monitoring at Seedling Stage of Winter Wheat

From the sowing stage to the seedling stage of winter wheat, the water demand accounts for 20% of the total water demand during the whole growth period. Droughts will influence seed germination and seedling emergence [64], causing a direct impact on the yield. The Huaibei Plain in Anhui Province is dry with little rainfall during the winter, and droughts occur easily, which has garnered attention. Research on the soil moisture and droughts of winter wheat has often focused on the periods with a high water consumption, such as the period of seedling establishment and the elongation stage [65]. Thus, monitoring the droughts during the seedling stage of winter wheat is important for obtaining a successful future harvest. During the seedling stage of winter wheat, the leaf area index (LAI) is small, and the soil coverage is low; among spectral indexes, the correlation with the soil indexes is high, and the inversion accuracy is relatively high. Generally, the depth inversion based on optical remote sensing can invert the soil moisture in the surface layer very well, while agricultural drought monitoring focuses on the soil moisture in the root layer of crops. According to the research on winter wheat in the Huaibei Plain [40], the key factor that influences the yield of winter wheat in the lime concretion black soil area in the Huaibei Plain is the soil moisture in the 0–20 cm soil layer; for soil moisture and drought monitoring of the wheat field, the evaluation of the soil moisture in the 0–20 cm soil layer can basically meet the requirements for a drought evaluation. Based on the UAV upscaling correction, this study improved the inversion accuracy of the relative soil moisture at depths of 0–20 cm and 0–40 cm, showing that this method can be successfully implemented for agricultural drought monitoring. In this study, the remote sensing indexes of drought were used for the inversion of soil moisture at a depth of 0–20 cm, and the result was better than the inversion of soil moisture at a depth of 0–10 cm. The reason for this may be threefold: at this stage, the root system of winter wheat starts to grow, but the root density is relatively low; the soil moisture is greatly influenced by the real-time weather; and there is a small, time difference between field sampling and satellite transit, causing a reduction in the simulation accuracy [66]. This study presents a novel approach to agricultural drought monitoring, which leverages the synergy of real-time unmanned aerial vehicle (UAV) imagery and free, readily accessible medium- and high-resolution satellite imagery. This method avoids the necessity of comparing a vast amount of historical data, offering a timely, comprehensive, and high-resolution solution to agricultural drought monitoring.

There are still some shortcomings in this study, which require in-depth discussions. The formation process of agricultural drought is extremely complicated and is affected by vegetation, rainfall, and other environmental factors. The land surface temperature also has a great influence on the soil moisture, since it directly influences energy balance components and ground surface evapotranspiration. The soil moisture content decreases as the land surface temperature rises. Therefore, research has often employed sol-air temperature vegetation dryness indexes such as the temperature vegetation dryness index (TVDI) and the vegetation supply water index (VSWI) for the inversion of soil moisture. This study corrected a satellite model with the aid of UAV multi-spectral remote sensing. Since temperature monitoring was not provided for the UAVs during this test, no sol-air temperature spectral indexes were selected for the inversion of the satellite-based relative soil moisture model, which affected the model’s accuracy to a certain extent.

## 5. Conclusions

This study employed a local average method for upscaling and matched UAV images with satellite images, using RF and XGBoost algorithms to establish the inversion models for the relative soil moisture at different depths based on measured land surface data. This research demonstrated the following: (1) The XGBoost model achieved the highest accuracy for the relative soil moisture of winter wheat at different depths in the seedling stage. (2) Compared with the soil depth model before the upscaling correction, a UAV correction can significantly improve the inversion accuracy of satellite remote sensing for relative soil moisture. (3) On this basis, the graph of the agricultural drought grade of winter wheat during the seedling stage in the Huaibei Plain in Anhui Province was drawn. This research provides a scientific basis for guiding and studying the agricultural drought of winter wheat during the seedling stage by combining satellite data and UAV data with ground data, and by employing machine learning to obtain an integrated satellite–UAV monitoring method. Future studies will consider rainfall, vegetation, temperature, and evapotranspiration, as well as other factors that influence agricultural droughts, and establish a comprehensive drought-monitoring model.

## Figures and Tables

**Table 1:** Remote sensing indexes for monitoring agricultural drought.

Indexes	Full Name	Calculation Formulas
NDVI	Normalized difference vegetation index	(IR − R)/(NIR + R)
OSAVI	Optimized soil adjusted vegetation index	1.16 (NIR − R)/(NIR + R + 0.16)
PDI	Perpendicular drought index	$(R+m\;\mathrm{NIR})/\sqrt{1+m^{2}}$
NDWI	Normalized difference water index	(NIR − G)/((NIR + G))
SAVI	Soil adjusted vegetation index	SAVI = (1 + L) × (NIR − R)/(NIR + R + L)
SAVI2	Soil adjusted vegetation index 2	NIR/(B + b/a)
DVI	Deviation vegetation index	NIR − R
EVI	Enhanced vegetation index	2.5 (NIR − R)/(1 + NIR + 6R − 7.5B)
RVI	Ratio vegetation index	NIR1/R
TVI	Triangular vegetation index	0.5 [120 (NIR − G) − 200 (R − G)]

Note: B, G, R, and NIR are blue, green, red, and near-infrared reflectance, respectively; m denotes the slope of the soil line obtained through linear regression of the lower edge of the triangle in the two-dimensional spectral eigenspaces of red light and near-infrared; L is a background adjustment factor for the cover, which was set to 0.5; and a and b denote the coefficients of the soil line, which were set to 1.0 and 0.5, respectively.

**Table 2:** Precision of inversion model for soil relative humidity at different depths.

Model	Depth/cm	Before The Upscaling Correction		After the Upscaling Correction	
		R^2^	RMSE (%)	R^2^	RMSE (%)
RF	0~10	0.4202	6.3264	0.4812	5.7113
	10~20	0.4511	5.4521	0.5036	4.5925
	20~40	0.3859	5.8890	0.4287	5.0533
	0~20	0.4602	5.2537	0.5407	4.4246
	0~40	0.4067	6.0221	0.4502	5.3914
XGBoost	0~10	0.4652	5.5759	0.5724	4.5383
	10~20	0.5004	5.1284	0.6402	4.0134
	20~40	0.4518	5.4983	0.5248	4.8602
	0~20	0.5519	4.8031	0.6863	3.8823
	0~40	0.4602	4.9235	0.5613	4.2846

## Data Availability

The research project is in progress, and the authors can be contacted for access to the data.

## References

[1] Trenberth K.E., Dai A., Van Der Schrier G., Jones P.D., Barichivich J., Briffa K.R., Sheffield J. (2014). Global warming and changes in drought. Nat. Clim. Chang..

[2] Tadesse T., Brown J.F., Hayes M.J. (2005). A new approach for predicting drought-related vegetation stress: Integrating satellite, climate, and biophysical data over the US central plains. ISPRS J. Photogramm. Remote Sens..

[3] Dalezios N., Blanta A., Spyropoulos N. (2012). Assessment of remotely sensed drought features in vulnerable agriculture. Nat. Hazards Earth Syst. Sci..

[4] Jiao W., Wang L., McCabe M.F. (2021). Multi-sensor remote sensing for drought characterization: Current status, opportunities and a roadmap for the future. Remote Sens. Environ..

[5] Lee H., Romero J., Core Writing Team (2023). Climate Change 2023: Synthesis Report, Summary for Policymakers. Contribution of Working Groups I, II and III to the Sixth Assessment Report of the Intergovernmental Panel on Climate Change.

[6] Dai A.G. (2011). Drought under global warming: A review. Wiley Interdiscip. Rev. Clim. Chang..

[7] Lloyd-Hughes B. (2014). The impracticality of a universal drought definition. Theor. Appl. Climatol..

[8] Wilhite D.A., Glantz M.H. (1985). Understanding: The drought phenomenon: The role of definitions. Water Int..

[9] Han D., Wang P.X., Zhang Y., Tian H.R., Zhou X.J. (2021). Progress of Agricultural Drought Monitoring and Forecasting Using Satellite Remote Sensing. Smart Agric..

[10] Sun H., Chen Y.H., Sun H.Q. (2012). Comparisons and classification system of typical remote sensing indexes for agricultural drought. Trans. Chin. Soc. Agric. Eng..

[11] West H., Quinn N., Horswell M. (2019). Remote sensing for drought monitoring & impact assessment: Progress, past challenges and future opportunities. Remote Sens. Environ..

[12] Choi M., Jacobs J.M., Anderson M.C., Bosch D.D. (2013). Evaluation of drought indices via remotely sensed data with hydrological variables. J. Hydrol..

[13] Yao Y., Chen X., Qian J. (2019). Advance in agricultural drought monitoring using remote sensing data. Spectrosc. Spectr. Anal..

[14] Fathololoumi S., Vaezi A.R., Alavipanah S.K., Ghorbani A., Biswas A. (2020). Comparison of spectral and spatial-based approaches for mapping the local variation of soil moisture in a semi-arid mountainous area. Sci. Total Environ..

[15] Liu X., Zhu X., Pan Y., Li S., Liu Y., Ma Y. (2016). Agricultural drought monitoring: Progress, challenges, and prospects. Geogr. Sci..

[16] Zheng M., Liu Z., Xu Z., Li J., Sun J. (2024). Research Progress of Soil Moisture Estimation Based on Microwave Remote Sensing. Acta Pedol. Sin..

[17] Zhang Z., Wang D., Wang G., Qiu J., Liao W. (2019). Use of SMAP soil moisture and fitting methods in improving GPM estimation in near real time. Remote Sen..

[18] Dorbu F., Hashemi-Beni L. (2024). Detection of Individual Corn Crop and Canopy Delineation from Unmanned Aerial Vehicle Imagery. Remote Sens..

[19] Jin Y., Wu X., Zhen W., Cui X., Chen L., Qie Z. (2024). UAV Multispectral Remote Sensing Inversion of Soil Moisture Content Based on Window Size Optimization of Spectral Information at Sampling Points. Trans. Chin. Soc. Agric..

[20] Kanaskie C.R., Routhier M.R., Fraser B.T., Congalton R.G., Ayres M.P., Garnas J.R. (2024). Early Detection of Southern Pine Beetle Attack by UAV-Collected Multispectral Imagery. Remote Sens..

[21] Khormizi H.Z., Malamiri H.R.G., Ferreira C.S.S. (2024). Estimation of Evaporation and Drought Stress of Pistachio Plant Using UAV Multispectral Images and a Surface Energy Balance Approach. Horticulturae.

[22] Wienhold K.J., Li D., Fang Z.N. (2024). Precision Irrigation Soil Moisture Mapper: A Thermal Inertia Approach to Estimating Volumetric Soil Water Content Using Unmanned Aerial Vehicles and Multispectral Imagery. Remote Sens..

[23] Sun J., Zhang D., Hou Y. (2021). Multi-source Remote Sensing Data Cooperates to Retrieve Forest Surface Soil Moisture. Remote Sens. Technol. Appl..

[24] Li X., Zhu C., Fu Z., Yan H., Peng Y., Zheng Y. (2020). Rapid Detection of Soil Moisture Content Based on UAV Multispectral Image. Spectrosc. Spectr. Anal..

[25] Feng S., Liang X., Fan F., Wang S., Wu J. (2020). Monitoring of Farmland Soil Moisture Based on Unmanned Aerial Vehicle Multispectral Data. South. China Norm. Univ. Nat. Sci. Ed..

[26] Zhu S., Cui N., Zhou J., Xue J., Wang Z., Wu Z., Wang M., Deng Q. (2023). Digital Mapping of Root-Zone Soil Moisture Using UAV-Based Multispectral Data in a Kiwifruit Orchard of Northwest China. Remote Sens..

[27] Cheng M., Sun C., Nie C., Liu S., Yu X., Bai Y., Liu Y., Meng L., Jia X., Liu Y. (2023). Evaluation of UAV-based drought indices for crop water conditions monitoring: A case study of summer maize. Agric. Water. Manag..

[28] Alexopoulos A., Koutras K., Ali S.B., Puccio S., Carella A., Ottaviano R., Kalogeras A. (2023). Complementary use of ground-based proximal sensing and airborne spaceborne remote sensing techniques in precision agriculture: A systematic review. Agronomy.

[29] Zhao F., Wu X., Wang S. (2020). Object-oriented vegetation classification method based on UAV and satellite image fusion. Procedia. Comput. Sci..

[30] Zhang S., Zhao G., Lang K., Su B., Chen X., Xi X., Zhang H. (2019). Integrated satellite, unmanned aerial vehicle (UAV) and ground inversion of the SPAD of winter wheat in the reviving stage. Sensors.

[31] Xi X., Zhao G.X., Gao P., Cui K., Li T. (2019). Inversion of Soil Salinity in Binhai Winter Wheat Growing Area Based on Sentinel satellite and UAV Multi-Spectrum: A Case Study of Kenli Area in the Yellow Triangle. Sci. Agric. Sin..

[32] Zhao L., Shi Y., Liu B., Hovis C., Duan Y., Shi Z. (2019). Finer classification of crops by fusing UAV images and Sentinel-2A data. Remote Sens..

[33] Mazzia V., Comba L., Khaliq A., Chiaberge M., Gay P. (2020). UAV and machine learning based refinement of a satellite-driven vegetation index for precision agriculture. Sensors.

[34] Jiang J., Atkinson P.M., Chen C., Cao Q., Tian Y., Zhu Y., Liu X., Cao W. (2023). Combining UAV and Sentinel-2 satellite multi-spectral images to diagnose crop growth and N status in winter wheat at the county scale. Field Crop Res..

[35] Bai X., Wang Y., Jin J., Ning S., Wang Y., Wu C. (2020). Spatio-temporal evolution analysis of drought based on cloud transformation algorithm over Northern Anhui Province. Entropy.

[36] Fang Y., Zhu Y., Lyu H., Wang Z., Pan Y., Xu H. (2023). Analyzing Drought Variation in Winter Wheat Growing Season in the Huaibei Plain. Irrig. Drain..

[37] Cui Y., Jiang S., Feng P., Jin J., Yuan H. (2018). Winter Wheat Evapotranspiration Estimation under Drought Stress during Several Growth Stages in Huaibei Plain, China. Water.

[38] Ren X., Xu G., Liu Y., Yang X. (2018). Characteristics of Spatiotemporal Variation of Climate in Anhui Province in Recent 56 Years. Res. Soil Water Conserv..

[39] Zhou Y., Zhou P., Jin J., Wu C., Cui Y., Zhang Y., Tong F. (2022). Drought identification based on Palmer drought severity index and return period analysis of drought characteristics in Huaibei Plain China. Environ. Res..

[40] Li D., Sun Y., Sun Y. (2017). Study on Scale Drought Index of Winter Wheat Growing Period in Sandy Ginger Black Soil in Northern Anhui Province. Triticeae Crops.

[41] Yuan H., Tang G., Yuan X., Zhao H. (2019). Analysis of Soil Moisture Characteristic Curve of No-till Farmland Based on Accelerated Genetic Algorithm. Water Sav. Irrig..

[42] Ali S., Henchiri M., Yao F., Zhang J. (2019). Analysis of vegetation dynamics, drought in relation with climate over South Asia from 1990 to 2011. Environ. Sci. Pollut. Res..

[43] Ghulam A., Qin Q., Zhan Z. (2007). Designing of the perpendicular drought index. Environ. Geol..

[44] Hazaymeh K., Hassan Q.K.J. (2017). A remote sensing-based agricultural drought indicator and its implementation over a semi-arid region, Jordan. Arid. Land.

[45] Li X., Ding J. (2015). Soil Moisture Monitoring Based on Measured Hyperspectral Index and HSI Image Index. Trans. Chin. Soc. Agric. Eng..

[46] Carranza C., Nolet C., Pezij M., van der Ploeg M. (2021). Root zone soil moisture estimation with Random Forest. J. Hydrol..

[47] Xia C., Jiang Y., Zhang X., Sha Y., Cui S., Mi G., Gao Q., Zhang Y. (2023). Estimation of soil organic matter in maize field of black soil area based on UAV hyperspectral image. Spectrosc. Spectr. Anal..

[48] Nguyen T.T., Ngo H.H., Guo W., Chang S.W., Nguyen D.D., Nguyen C.T., Zhang J., Liang S., Bui X.T., Hoang N.B. (2022). A low-cost approach for soil moisture prediction using multi-sensor data and machine learning algorithm. Sci. Total Environ..

[49] Song Y., Fang S., Liang H., Ke L. (2017). Comparison and application of agricultural drought indexes based on MODIS data. Remote Sens. Land Resour..

[50] Liu Y., Xia X., Yao L., Jing W., Zhou C., Huang W., Li Y., Yang J. (2020). Downscaling satellite retrieved soil moisture using Regression tree-based machine learning algorithms over Southwest France. Earth Space Sci..

[51] Yang J., Huang X. (2021). The 30 m annual land cover dataset and its dynamics in China from 1990 to 2019. Earth Syst. Sci. Data.

[52] (2015). Grade of Agricultural Drought.

[53] Sun L., Wang F., Wu Q. (2010). Drought monitoring by remote sensing in winter-wheat-growing area of China. Trans. Chin. Soc. Agric. Eng..

[54] Tong D., Bai Y., Zhang S., Liu Q., Yang J. (2020). Applicability of Drought Severity Index (DSI) in drought remote sensing monitoring in Shandong province. Chin. Agric. Agrometeorol..

[55] Monteiro A.T., Arenas-Castro S., Punalekar S.M., Cunha M., Mendes I., Giamberini M., Marques Da Costa E., Fava F., Lucas R. (2024). Remote sensing of vegetation and soil moisture content in Atlantic humid mountains with Sentinel-1 and 2 satellite sensor data. Ecol. Indic..

[56] Luo L., Li Y., Guo F., Huang Z., Wang S., Zhang Q., Zhang Z., Yao Y. (2023). Research on robust inversion model of soil moisture content based on GF-1 satellite remote sensing. Comput. Electron. Agric..

[57] Sunny D.S., Islam K.M.A., Mullick M.R.A., Ellis J.T. (2022). Performance study of imageries from MODIS, Landsat 8 and Sentinel-2 on measuring shoreline change at a regional scale. Remote Sens. Appl. Soc. Environ..

[58] Zhang C., Xiao X., Wang X., Qin Y., Doughty R., Yang X., Meng C., Yao Y., Dong J. (2024). Mapping wetlands in Northeast China by using knowledge-based algorithms and microwave (PALSAR-2, Sentinel-1), optical (Sentinel-2, Landsat), and thermal (MODIS) images. Environ. Manag..

[59] Zhu X., Cai F., Tian J., Williams T.K.-A. (2018). Spatiotemporal Fusion of Multisource Remote Sensing Data: Literature Survey, Taxonomy, Principles, Applications, and Future Directions. Remote Sens..

[60] Emilien A., Thomas H., Thomas C. (2021). Corrigendum to ‘UAV & satellite synergies for optical remote sensing applications: A literature review’. Sci. Remote Sens..

[61] Huang S., Miao Y., Yuan F., Martin G., Yao Y., Cao Q., Wang H., Victoria L.W., Georg B. (2017). Potential of RapidEye and WorldView-2 Satellite Data for Improving Rice Nitrogen Status Monitoring at Different Growth Stages. Remote Sens..

[62] Li Y., Chang C., Wang Z., Zhao G. (2023). Upscaling remote sensing inversion and dynamic monitoring of soil salinization in the Yellow River Delta, China. Ecol. Indic..

[63] Hassan-Esfahani L., Ebtehaj A.M., Torres-Rua A., McKee M. (2017). Spatial Scale Gap Filling Using an Unmanned Aerial System: A Statistical Downscaling Method for Applications in Precision Agriculture. Sensors.

[64] Xu H., Huang J., Zhu Y., Lyu H., Liu Y., Wang Z. (2021). Spatiotemporal Variation of Soil Water Content over Winter Wheat Fields in Huaibei Plain. Irrig. Drain..

[65] Wang S., Li Q., Wang H., Du X., Gao L. (2021). A Winter Wheat Drought Index based on TROPOMI Solar-Induced Chlorophyll Fluorescence. Remote Sens. Technol. Appl..

[66] Zhang X., Liu H., Yan Y., Tang R., Zhang Y. (2021). Retrieval of Surface Soil Water Content Using Remote Sensing in Incorporation with Phenological Traits of Crops. Irrig. Drain..

